# Changes in automated external defibrillator use and survival after out-of-hospital cardiac arrest in the Nijmegen area

**DOI:** 10.1007/s12471-018-1162-9

**Published:** 2018-10-02

**Authors:** J. Nas, J. Thannhauser, J. J. Herrmann, K. van der Wulp, P. M. van Grunsven, N. van Royen, M. J. de Boer, J. L. Bonnes, M. A. Brouwer

**Affiliations:** 1 Department of Cardiology, Radboud University Medical Center, Nijmegen, The Netherlands; 2 Regional Ambulance Service Gelderland-Zuid, Nijmegen, The Netherlands

**Keywords:** Out-of-hospital cardiac arrest, Survival, Automated external defibrillator

## Abstract

**Purpose:**

Out-of-hospital cardiac arrests (OHCAs) are a major healthcare problem. Over the years, several initiatives have contributed to more lay volunteers providing cardiopulmonary resuscitation (CPR) and increased use of automated external defibrillators (AEDs) in the Netherlands. As part of a quality and outcomes program, we registered bystander CPR, AED use and outcome in the Nijmegen area.

**Methods:**

Prospective resuscitation registry with a study cohort of non-traumatic OHCA cases from 2013–2016 and historical controls from 2008–2011. In line with previous reports, we studied patients transported to the hospital (Radboudumc, Nijmegen, the Netherlands) and excluded arrests witnessed by the emergency medical service (EMS). Primary outcomes were return of spontaneous circulation (ROSC) and survival to discharge.

**Results:**

In the study cohort (*n* = 349) the AED was attached more often than in the historical cohort (*n* = 180): 46% vs. 23% and the proportion of bystander CPR was higher: 78% vs. 63% (both *p* < 0.001). A higher proportion of patients received an AED shock (39% vs. 15%, *p* < 0.001) and the number of required shocks by the EMS was lower (2 vs. 4, *p* = 0.004). Survival to discharge was higher (47% vs. 33%, *p* = 0.002) without differences in ROSC. The survival benefit was restricted to patients with a shockable initial rhythm. In both cohorts, bystander CPR and AED use were independently associated with survival.

**Conclusion:**

In patients admitted after OHCA, survival to discharge has markedly improved to 40–50%, comparable with other Dutch registries. As increased bystander CPR and the doubled use of AEDs seem to have contributed, all civilian-based resuscitation initiatives should be encouraged.

## What’s new

- To improve awareness and performance of early cardiopulmonary resuscitation (CPR), a network of first responders (i. e. firemen/police), national awareness campaigns and local initiatives to improve lay person CPR have been employed, including strategies to ensure 24/7 automated external defibrillator (AED) accessibility.
- We found higher rates of bystander CPR and AED use in our current cohort of cardiac arrest patients than in a historical cohort, as well as higher survival.
- Our findings support continued efforts to increase bystander CPR and AED use.

## Introduction

Out-of-hospital cardiac arrests (OHCAs) are a major healthcare problem with dismal survival rates [1]. In the Netherlands, about 300 OHCAs occur each week [2]. Internationally, survival to discharge varies between 5%–25% and our national survival rates are relatively favourable with an average of 23% among all adults treated for OHCA [3, 4], and 40–50% in patients transported to the hospital [5, 6].

Worldwide, there have been many initiatives to improve survival [7, 8], which are summarised in a framework called the Chain of Survival [9]. In the pre-hospital setting, interventions are mainly aimed towards improving bystander cardiopulmonary resuscitation (CPR) and facilitating earlier defibrillation [10–12]. An important development is increased availability and use of automated external defibrillators (AEDs) [13–16]. Initially, AEDs were mainly used by first responder units, such as fire and police services, which are dispatched alongside the ambulance to a suspected OHCA [17]. More recent developments involve dispatching lay responders to find and bring the nearest AED to the scene of the OHCA, using a text message based alert system [18–21]. Recruitment of lay responders may facilitate early defibrillation, a key determinant of survival in case of a shockable initial rhythm [22].

In our region of Nijmegen several initiatives have been undertaken to improve pre-hospital care by lay volunteers over the past years. On a community basis, lay persons have been trained to perform CPR and use the AED. Civilians, without interference of professional healthcare providers, have set up a network that registers AEDs and guarantees maintenance. In addition, this civilian network promotes registration in a national centralised text message based alert system of both trained volunteers and available AEDs.

As part of an initiative to start up a quality and outcomes program in our region, we initiated an evaluation of our local OHCA characteristics and outcomes, as collected in our prospective resuscitation registry [23]. The primary aim was to study bystander CPR, AED use and survival over the past years and to put these in perspective with data on historical controls from this registry.

## Methods

### Patient population

From our regional prospective resuscitation registry, comprising urban, suburban and rural areas, we selected a study cohort and historical controls. The former consists of non-traumatic OHCA cases (age ≥18 years) resuscitated by the emergency medical services (EMS) between 2013–2016. For reasons of comparability with previous publications, we focussed on patients transported to a single hospital (Radboudumc Nijmegen) [5, 6] and excluded emergency medical service (EMS) witnessed arrests [13]. The latter consists of patients resuscitated between 2008–2011 [23], who fulfilled the same inclusion and exclusion criteria. Given the observational design of the study, it was not necessary to obtain written informed consent according to the Dutch Act on Medical Research involving Human Subjects.

### Emergency medical service

The Dutch EMS system is activated by calling 112. Paramedics will give instructions to the caller to initiate basic life support (BLS), and usually two ambulances are dispatched to the location of the emergency. Ambulances are staffed by a driver and a paramedic, who are professionally trained to perform advanced life support. CPR was performed according to the prevailing guidelines at the time of the OHCA [10–12].

### Data collection

Demographic, clinical and arrest characteristics were collected using EMS and hospital records. Variables were defined according to the Utstein style definitions [24]. Location was divided into public or non-public [25]. Outcome data were collected using hospital records and information from municipal registries.

### Patient cohorts

Patients were categorised as follows. The study cohort included patients resuscitated between January 2013 and December 2016, while the patients in the historical control cohort were resuscitated between April 2008 and January 2011.

### Outcome measures

The primary outcome was survival to hospital discharge. The secondary outcome was sustained ROSC, defined as hospital transportation with ROSC. We furthermore reported on any ROSC in the field, ROSC at the emergency department (ED) and 24-hour survival.

### Statistical analysis

Data were presented and compared as described previously [23]. Analyses were performed separately for 2008–2011 and 2013–2016. To evaluate whether AED use and bystander CPR were associated with survival, multivariate binary logistic regression analysis was performed. All factors that were univariately associated with survival (*p* < 0.10) were included. Results were reported as adjusted odds ratios (aOR) and their 95% confidence intervals. A *p*-value of <0.05 was considered statistically significant for all analyses (SPSS Statistics v.22, IBM-Corp., Armonk, NY, USA).

## Results

We studied 529 patients, of whom 349 patients were resuscitated between 2013–2016 (study cohort) and 180 between 2008–2011 (historical controls). In the study cohort, a smaller proportion of the patients received amiodarone or epinephrine. Gender, age, response time and proportions of public location, bystander witnessed and initial shockable rhythm did not differ between the patient cohorts (Tab. 1).

**Table 1:** Baseline characteristics of both patient cohorts

	2008–2011 *n* = 180	2013–2016 *n* = 349	*P-*value
Male gender	131 (73)	266 (76)	0.39
Age (years)	64 (53–76)	64 (53–74)	0.74
Public location	71 (40)	156 (47)	0.12
Bystander witnessed	133 (79)	263 (79)	0.94
Bystander CPR	112 (63)	265 (78)	<0.001
Initial shockable rhythm	132 (74)	250 (74)	0.96
Shocked by EMS	136 (76)	202 (59)	<0.001
If yes, no. of shocks	4 (1–6)	2 (1–5)	0.004
EMS response time (minutes)	8 (6–10)	10 (5–12)	0.45
Amiodarone	61 (39)	86 (26)	0.004
Epinephrine	128 (81)	195 (58)	<0.001
AED attached	41 (23)	158 (46)	<0.001
AED shocked	27 (15)	135 (39)	<0.001

Values are presented as numbers (percentages) or medians (interquartile range)

*CPR* cardiopulmonary resuscitation, *EMS* emergency medical services, *AED* automated external defibrillator

### Bystander CPR and AED use

The proportion of patients who received bystander CPR was higher in the study cohort than in the historical cohort (78% vs. 63%, *p* < 0.001). The proportion of patients in whom an AED was attached was also higher (46% vs. 23%, *p* < 0.001). Furthermore, a higher proportion of patients received a shock by the AED (39% vs. 15%, *p* < 0.001), less patients received a shock by the EMS (59% vs. 76%, *p* < 0.001) and the median number of EMS shocks was lower in the study cohort (2 vs. 4, *p* = 0.004).

### Clinical outcomes

In the study cohort, survival to discharge was higher than in the historical controls (47% vs. 33%, *p* = 0.002), while ROSC rates were similar (65% vs. 67%, *p* = 0.74). No significant differences were found in the other outcome parameters (Tab. 2). The survival benefit was restricted to patients with a shockable initial rhythm (58% vs. 41%, *p* = 0.002 in shockable and 13% vs. 9%, *p* = 0.51 in non-shockable rhythms).

**Table 2:** Clinical outcomes of both patient cohorts

	2008–2011 (*n* = 180)	2013–2015 (*n* = 349)	*P*-value
*ROSC*			
Any field	139 (77)	253 (73)	0.28
Sustained	120 (67)	221 (65)	0.74
At emergency department	121 (67)	233 (67)	0.99
*Survival*			
24-hour	102 (58)	224 (65)	0.14
To hospital discharge	60 (33)	165 (47)	0.002

Values are presented as numbers (percentages)

*ROSC* return of spontaneous circulation

### Factors associated with survival to discharge

In the study cohort, we found that younger age, male gender, public location, bystander witnessed, shockable initial rhythm, bystander CPR and AED use were associated with survival. Bystander CPR (aOR 2.83, [1.45–5.51], *p* = 0.002) and AED shocks (aOR 2.36 [1.40–4.00], *p* = 0.001) were independently associated with survival. In the historical cohort, younger age, female gender, bystander CPR, shockable initial rhythm and AED shocks were associated with survival. Bystander CPR (aOR 2.45 [1.14–5.27], *p* = 0.02) and AED shocks (aOR 3.18 [1.25–8.09], *p* = 0.02) were independently associated with survival.

## Discussion

In this prospective registry of OHCA patients, we observed higher rates of bystander CPR and a doubled use of AEDs in the 2013–2016 cohort when compared with historical controls from 2008–2011, along with an increase in survival from 33 to 47%. In both cohorts, bystander CPR and AED use were independently associated with survival. These findings underscore the importance of BLS and AED use, and support the national campaign of the Dutch Heart Foundation to improve regional coverage by civilian BLS volunteers and AEDs.

### Resuscitation outcome in the Netherlands

In 2015, the Dutch Heart Foundation initiated the Working Group Cardiac Arrest Netherlands (weCAN). This working group aimed to unite experts in the field (from Amsterdam, Utrecht, Maastricht and Nijmegen) and report on outcomes and recent developments in OHCA. The main finding was that overall survival increased from 9 to 23% in recent years [3]. Notably, this concerns survival of all OHCA patients, whereas we now focus on patients who were transported to the hospital, similar to other recent publications on this subject [5, 6]. We found that survival rates in recent years increased from 33 to 47%. In Maastricht, a survival rate of 46% was found, compared with a survival rate of 43% in Leiden [5, 6]. Overall, baseline characteristics are comparable among these three cohorts. However, in Leiden there were lower proportions of shockable initial rhythms (61% vs. 74% in our 2013–2016 cohort) and bystander CPR (51% vs. 78% in our cohort). As these are both strong predictors of survival, this might explain our slightly higher survival rates.

### Impact of the AED

Since its development, the AED has been extensively investigated with regard to safety, costs and its effect on survival [13–16, 26]. In recent years, AEDs have become increasingly available to first responders and bystanders, resulting in improved outcome [17, 27]. In our region, a civilian-based foundation has developed several initiatives to improve resuscitation education and establish a network of AEDs throughout the municipality. This foundation acquires, registers and maintains AEDs and is funded by private sponsoring. Members of the foundation also promote and facilitate registration of BLS providers and AEDs in a nation-wide, text message based civilian alert system. In addition to these continuing and expanding efforts throughout our community, regional government has contributed as well, by moving indoor AEDs to outside locations. This increases the availability of these AEDs from exclusively during business hours to 24/7. These AEDs were also registered in text message alert systems.

In the present study, we found an increase in bystander CPR and AED use. We also observed that survival in our region has significantly increased over the last years. In both cohorts, AED use was independently associated with survival. The fact that the improvement in survival was restricted to patients with a shockable rhythm is in line with results from a previous meta-analysis [26]. These observations support the concept that increased AED use contributed to our improved outcomes [13–15]. At present, our database does not systematically capture the use of the text message systems, whether the AEDs used were registered AEDs and whether BLS was performed by trained volunteers. This limits conclusions with regard to the contribution of each of the individual factors.

### ROSC vs. survival to discharge

ROSC rates were high in 2008–2011 and have remained high, yet more patients now survive until discharge. Faster restoration of cerebral perfusion may account for this, as patients treated with an AED have a shorter CPR duration and, thus, shorter periods of cerebral hypoxia, a major determinant of survival after OHCA [28]. Even though we did not have the exact times to ROSC, several findings point towards shorter CPR duration. Firstly, we found decreased use of epinephrine and amiodarone as well as a lower number of EMS shocks in the study cohort. Secondly, in the patients with an AED attached (46% study cohort vs. 23% historical control cohort), the proportion receiving a shock was markedly higher (Tab. 1): 85% in the former versus 66% in the latter. This may be related to earlier attachment of the AED, as the chances of finding a shockable rhythm increase with shorter arrest durations [29]. Thirdly, a larger number of patients with a shockable initial rhythm did not receive a shock by the EMS in the 2013–2016 cohort. Notably, all these patients received an AED shock, implying earlier termination of the shockable rhythm. We hypothesise that these early benefits of the AED are sustained throughout the post-resuscitation period and ultimately result in increased survival. However, despite these plausible clues, we cannot firmly objectify earlier AED attachment in lack of data on AED time intervals. Our future registration will also focus on these intervals, to further objectify benefits achieved with lay rescuers.

### Implications

Our findings underscore the importance of increased rates of bystander CPR and AED use, and support all initiatives to increase awareness for early BLS and use of an AED in case of a cardiac arrest. Although part of the observed increase in bystander CPR will be related to increased awareness through national media campaigns (‘6-minute zones’, Dutch Heart Foundation, https://www.hartstichting.nl/reanimatie/6-minutenzone), our local initiative might have contributed as well. Importantly, our findings have a descriptive nature, and should not be interpreted as the result of a local intervention.

Previous publications have shown the value of a text message based alert system in the Netherlands. This system alerts registered volunteers to go to the site of OHCA, directly or via the nearest AED [18–21]. In recent years, the number of volunteers registered in these systems in the Netherlands increased from 41,805 in 2012 to 95,212 in 2015. The number of registered AEDs also increased, from 5,275 to 7,945. Appreciating the potential benefit of civilian-based initiatives in terms of higher availability of AEDs and more trained volunteers, we strongly support all innovations in this context and encourage healthcare providers to subscribe (www.hartslagnu.nl and www.hartveiligwonen.nl). Furthermore, we feel that our initiative to increase 24/7 accessibility of AEDs could be a promising intervention to increase early AED use.

### Limitations

This is an observational cohort study, and although the historical controls are part of the same prospective registry, not all differences that develop over time will have been captured which may have affected our results. In particular, even though the ratio public/non-public and median EMS response times did not differ between cohorts, we cannot exclude small differences with regard to the location of the arrest. As for underlying aetiology (presumed cardiac, traumatic, other) no differences were observed between cohorts. Importantly, the vast majority of other baseline characteristics did not differ, except for bystander CPR and AED use. Rates of in-field termination and hospital transportation have been stable over the years as well [23]. Thus, although unmeasured variables may have influenced outcome, this will only have partly influenced the magnitude of the observed survival benefit, and it is unlikely that this has affected the observed direction of the effect on survival. For reasons of comparability [5, 6], our data are restricted to OHCA patients transported to the hospital, which should be noted when comparing survival rates with studies that include all OHCA victims [30]. Due to logistical issues, no data were available for 2012.

## Conclusion

In patients admitted after OHCA, survival to discharge has markedly improved to about 40–50%, comparable with other Dutch registries. Increased bystander CPR and the doubled use of AEDs in a setting of civilian-driven initiatives seem to have contributed and should therefore be encouraged.
